# Cerebrovascular involvement in hereditary spherocytosis: observational cohort and case-control MRI study

**DOI:** 10.1186/s13023-025-04121-4

**Published:** 2025-11-25

**Authors:** Renzo Manara, Marcella Contieri, Giovanni Librizzi, Teresa Palma, Saverio Scianguetta, Carmine Perna, Annalisa Valentina Villani, Sofia Maria Rosaria Matarese, Margherita Luciano, Alessia Amodio, Maria Agnese Pirozzi, Raffaella Colombatti, Claudia Santoro, Mario Cirillo, Francesco di Salle, Fabrizio Esposito, Silverio Perrotta, Immacolata Tartaglione

**Affiliations:** 1 Dept of Neuroscience, Neuroradiology, University of Padova, Padova, Italy; 2 Dipartimento della Donna, Del Bambino e di Chirurgia Generale e Specialistica, Università Degli Studi della Campania “Luigi Vanvitelli”, Napoli, Italy; 3 Department of Advanced Medical and Surgical Sciences, Università Degli Studi della Campania “Luigi Vanvitelli”, Napoli, Italy; 4 Dipartimento della Salute della Donna E Del Bambino, Università Degli Studi di Padova, Padova, Italy; 5 Dipartimento di Medicina E Chirurgia, Scuola Medica Salernitana, Università di Salerno, Salerno, Italy

**Keywords:** Hereditary spherocytosis, Brain MRI and MR-Angiography, Moyamoya syndrome, Cerebrovascular disease

## Abstract

**Background:**

Anecdotal Literature regarding hereditary spherocytosis, a rare hemolytic anemia, points to an early cerebrovascular involvement that would imply early strict patients’ monitoring and management. However, this issue is under-investigated.

**Methods:**

Clinical history regarding cerebrovascular events in the young ( < 55 years) was investigated in a referral Center for hereditary spherocytosis (190 patients; mean-age 23.5 ± 17.4 years; range 1–77; 93 females). By means of 3T-MR scanner, intracranial artery stenoses, aneurysms, brain infarctions, vascular-like white matter lesions and signs of sinus thrombosis were searched in 69 adult patients (mean-age 36.5 ± 16.3 years, range 14–76, 38 females) and in 56 healthy subjects (mean-age 34 ± 10.8 years, range 17–66, 36 females). Laboratory and clinical data, including splenectomy, were collated.

**Results:**

Among patients no cerebrovascular events occurred before the age of 55, while among their first-degree relatives, sudden death of unknown cause occurred in three subjects, two of them not affected by hereditary spherocytosis. In the MR-subgroup, moya-moya or even intracranial stenoses were not observed, while intracranial aneurysm (11.6% vs 8.9%), gray matter infarction (one patient), previous venous thrombosis (one patient) and white matter lesion (49.3% vs 50%) prevalence rates did not differ significantly between patients and controls. White matter changes did not differ also in terms of number or size and were not associated with laboratory or clinical findings.

**Conclusions:**

In spite of an evocative case-report-based Literature, early cerebrovascular involvement in hereditary spherocytosis does not seem to differ significantly from the general population. A strict clinical and MR monitoring in neurological asymptomatic patients seems therefore not recommended.

## Introduction

Hereditary spherocytosis (HS, Minkowski-Chauffard disease, #182900) is a genetically determined disease which affects red blood cells leading to chronic hemolytic anemia [1]. It is characterized by the presence of splenomegaly, jaundice and spherocytes in peripheral blood smears [2] with a variable degree of anemia. Patients with the most severe forms require transfusions since early childhood for preventing multi-organ failure and cognitive impairment. In these patients, splenectomy is an effective therapeutic option, as it may result in transfusion-independence. However, splenectomy may favor a hypercoagulative state with increased risk of thrombosis [3, 4] in all districts, including the brain. So far, studies on cerebrovascular involvement in HS due to anemia and splenectomy are lacking and this issue remains poorly investigated.

Besides, similarly to sickle cell disease, some HS children have been diagnosed with moyamoya disease [5–7], a rare cerebrovascular disorder characterized by progressive bilateral intracranial artery steno-occlusion associated with a compensatory rich intraparenchymal net of arteriolar collaterals prone to ischemic and hemorrhagic strokes [7, 8]. As the pertaining Literature is limited to case reports, prevalence and predisposing factors to moya-moya disease remain unknown.

Finally, studies on other genetically-determined hemolytic anemias have reported increased incidence of intracranial aneurysms [9, 10], silent infarcts or vascular like white matter changes [11]. All these aspects may lead to early potentially devastating cerebrovascular events (e.g. ischemic strokes, subarachnoid hemorrhages) or in progressive cognitive impairment [12] prompting for a systematic/scheduled monitoring of HS patients since the early phases of the disease. However, to date, the Literature in HS is extremely scarce and the prevalence and type of brain parenchymal and vascular involvement in HS patients and their association with clinical, laboratory, and treatment findings remain still undefined.

In order to improve our knowledge regarding cerebrovascular involvement in HS we structured the present study in two parts: first we investigated by phone interview and clinical file examination, all cerebrovascular clinical events in the young in our whole cohort of HS patients including their first-degree relatives. Concurrently, we searched by means of 3T-MR scanner all brain parenchymal and intracranial vascular changes in a large subgroup of adult patients, comparing their findings with those of a proper healthy control group.

## Materials and methods

### Subjects

The database of the Pediatric Hematology and Rare Anemias of the AOU Policlinico “Luigi Vanvitelli” (ERN EuroBloodNet) was searched for all the Spherocytosis diagnosis that occurred over the past 10 years and for the Spherocytosis patients in active follow-up. The diagnosis of HS was established based on family history, presence of nonimmune hemolytic anemia, and assessment of spherocyte morphology and was confirmed by abnormal incubated osmotic fragility test. Clinical charts review and phone interviews were carried out to detect patients’ and their first-degree relatives’ conditions, with special regard to any stroke, transient ischemic attack or sudden death.

Patients were invited to undergo brain Magnetic Resonance parenchymal and vascular (MR-angiography) investigations. Inclusion criteria for the imaging study were: diagnosis of HS and age ≥14 years. Exclusion criteria were: contraindications to MR, history of head trauma, neurosurgery, and concomitant neurological disease.

Clinical and laboratory data related to the disease history and severity were collected from clinical charts; laboratory parameters (see Table 1) were collected as a mean of the year previous to study enrolment. All data were acquired between December 2022 to December 2023.

**Table 1:** Baseline characteristics of subjects undergoing MRI

	All HS Patients	Splenectomized	Non-splenectomized	Controls
*N*	69	49	20	56
Mean-age, years	36.5±16.3	36.3±16.3	37.1±16.8	34.0±10.8
Female sex, *n* (%)	38 (55.1)	29 (59.1)	9 (45)	36 (64.2)
Mean-age at diagnosis, years	5.8±7.7	4.7±5.6	13.2±14.2	-
Mean Hb*, g/dL	13.5±1.6	13.8±1.7	12.9±1.4	NA
Reticulocytes, x10^6/uL	0.15±0.09	0.11±0.06	0.22±0.10	NA
History of Chronic Transfusions°, *n* (%)	27 (39.1)	27 (55.1)	0	-
On aspirin, n (%)	5 (7.2)	5 (10.2)	0 (0)	0 (0)
Platelet count, x10^3/uL	415±150	464±132	253±71	NA
Total Bilirubin, mg/dL	1.6±1.2	0.9±0.4	2.6±1.3	NA
Unconjugated bilirubin, mg/dL	1.2±1.1	0.6±0.3	2.1±1.3	NA
Lactate dehydrogenase, U/L	231.4±58.8	248.4±62.4	202.4±40.9	NA
Cholecystectomy, *n* (%)	27 (39.1)	22 (44.8)	5 (25)	0
Comorbidity, *n* (%)	18 (26)	14 (28.5)	4 (20)	0

Legend: Mean Hb* = Mean hemoglobin during the year prior to the MRI; ° = More than 8 transfusions/year for at least one year; na = not applicable; NA = not available

Controls were healthy volunteers mainly enrolled from patients’ relatives or entourage, in order to minimize environmental confounding factors on brain involvement. Their selection followed the same exclusion criteria used for the case group.

The study protocol was approved by the institutional review board and written informed consent was obtained from participants and/or their parents before any study procedure (Prot. 0034213/i date 11/11/2022).

*MRI and* MR-angiography

All exams were acquired on the same 3.0T MRI scanner (MAGNETOM Skyra, Siemens, Erlangen Germany) with a 20-channel head coil. The MR study protocol included:

1. 3D fluid attenuated inversion recovery (FLAIR, TR/TE/TI 5000/387/1800 ms; voxel-size 1*1*1 mm; echo-train length 278; field of view 230; acquisition-time 4 min 32 s). Axial, coronal and sagittal multiplanar reconstructions of the whole brain were obtained (slice thickness 3 mm without interslice gap).
2. 2D echo-planar diffusion-weighted imaging (DWI, TR/TE 9500/95 ms; acquisition matrix 128*128; slice thickness 2 mm; interslice gap 0 mm; field of view 256; b-value 1000 s/mm^2^; number of excitations 1; 30 non-collinear gradient directions; acquisition-time 6 min 31 s). Trace DWI images and apparent diffusion coefficient (ADC) maps were automatically generated.
3. 3D multi-slab Time of Flight MR-angiography investigating the intracranial carotid and vertebrobasilar arteries vertebral arteries, the intrapetrous and intracavernous internal carotid artery, the circle of Willis, the M1-M2 segments of the middle cerebral arteries, the pre- and post-communicating segments and the major branches of the anterior and posterior cerebral arteries, respectively (TR/TE 21/3.43 ms; voxel-size 0.6x0.6x0.7 mm; field of view 200 mm; number of slabs 120; number of partitions/slab 40; acquisition-time 3 min 34 s). Maximum intensity projection views were produced in the three orthogonal axes (12-view series each covering 180°) from artery MR angiography to allow the precise visualization of artery segments.
4. Phase contrast MR-venography covering all intracranial venous sinuses (TR/TE 80.55/10.6 ms; matrix 192x192; slice thickness 1 mm; field of view 192 mm; velocity-encoding 8 cm/s; acquisition-time 6 min 53 s). Three orthogonal maximum intensity projections of the MR-venography were automatically produced by the scanner and used for evaluation.

Brain and intracranial vessel MR examinations were visually assessed in consensus by three expert neuro-radiologists blinded to clinical data (RM, MC, FdS).

Artery stenosis and/or aneurysms were evaluated on MR-angiography (both partitions and maximum intensity projections were considered). The 5-year risk of rupture of aneurysm was assessed by means of the *Population, Hypertension, Age, Size, Earlier subarachnoid hemorrhage, and Site score* [13].

Venous sinus thrombosis was evaluated on MR venography: whenever a segment of the main venous sinuses appeared smaller than expected (e.g. anterior portion of the anterior sagittal sinus, right or left transverse sinuses), FLAIR reconstructions orthogonal to the sinus course (coronal and sagittal, respectively) were investigated to differentiate anatomical variants (e.g. hypo/aplasia) from partial/complete venous sinus thrombosis.

The white matter lesion (WML) load was evaluated on FLAIR images. According to the semi-quantitative Fazekas score [14, 15] assigning a value ranging from 0 to 3 based on lesion size, number and morphology. Lesion number and maximum diameter ( < 0.5 cm; 0.5–1.5 cm; > 1.5 cm) were also recorded. The size of the largest lesion was considered in statistical analysis. DWI was evaluated to differentiate acute from chronic vascular WML.

### Statistical analysis

Comparisons between groups were performed using the 2-tailed T-test, Mann-Whitney U-test and Chi-square tests (or the Fisher Exact test when required) for respectively normally distributed, ordinal and qualitative non-ordinal variables. The linear correlation between two variables was tested using the Spearman’s rho. Statistical significance was set at *p < 0.05*.

### Literature review

All published studies dealing with cerebrovascular involvement in HS patients were extensively searched in PubMed/Medline, EMBASE, and Google using “Hereditary Spherocytosis” associated with “Stroke”, “Transient ischemic attack”, “Moyamoya”,”Aneurysm”, “Silent infarct” or “White matter hyperintensities”. The retrieved articles were analyzed and non-pertinent studies were excluded. The search was thereafter extended to the references of the identified papers.

## Results

### Patients

One-hundred ninety patients (median-age 19 years, IQR 10.3–33 years; 10 older than 55 years; 93 (49.0%) females, 94 (49.5%) splenectomized) were recruited. Stroke or clinical cerebrovascular events before the age of 55 were not reported among our patients and their first-degree relatives. Death for unknown causes was reported in one 43-year-old male HS patient and two subjects not affected by hereditary spherocytosis at the age of 9 and 62.

Sixty-nine patients (mean-age 36.5 ± 16.3 years, range 14–76; 10 older than 55 years; 38 females) underwent MR-angiography, venography and conventional MRI. One hundred twenty-one did not participate to the MRI study because of age < 14 years (66 patients), confounding neurological comorbidities (e.g. history of severe head trauma or multiple sclerosis), claustrophobia or simply because not willing to participate further, for example, for the distance of the MR scanner located in Salerno, etc. (55 patients). Fifty-six healthy controls (mean-age 34 ± 10.8 years, range 17–66, 36 females) had the same MR protocol; they were briefly interviewed before the MRI as cerebrovascular risk factors (hypertension and cardiopathy) served as exclusion criterion. Patients and controls did not differ for age and sex (*p* = 0.98 and *p* = 0.30, respectively). Splenectomy was performed in 51/69 patients (74%; mean-age at splenectomy 13.7 ± 9.4 years). Main demographic, clinical and laboratory findings are reported in Table 1. History of therapy with acetylsalicylic acid was reported in 16/49 splenectomized patients (32.6%) were exposed to ASA right after splenectomy; the mean time of exposure was 1.5 ± 0.3 year.

Heterogeneous co-morbidities were found in 18/69 patients: rheumatoid arthritis (two), fibromyalgia (one), hypercholesterolemia (one), migraine (one), hypertension (four), gout (one), hypothyroidism (two), cognitive delay (one), panic attacks (one), multinodular goiter (two), mitral valve prolapse (one), psoriatic arthritis (one), thyroiditis (one), psoriasis (one), asthma (one), osteoporosis (one), mild aortic regurgitation (one), proteinuria (one), keratoconus (one), scoliosis (one).

No patient or control showed moyamoya disease or intracranial artery stenosis consistent with initial moyamoya disease. Aneurysms were found in 8/69 (11.6%) patients and 5/56 (8.9%) controls (*p* = 0.63; Chi-square test). In all patients and controls, the PHASES score was ≤5 with a rather low 5-year risk of rupture of aneurysm (≤1.3%).

A 30-year-old female HS patient showed asymptomatic lumen irregularities of left transverse and sigmoid venous sinuses consistent with previous partially recanalized thrombosis, though clinical history and neurological examination were unremarkable.

**Fig. 1:**
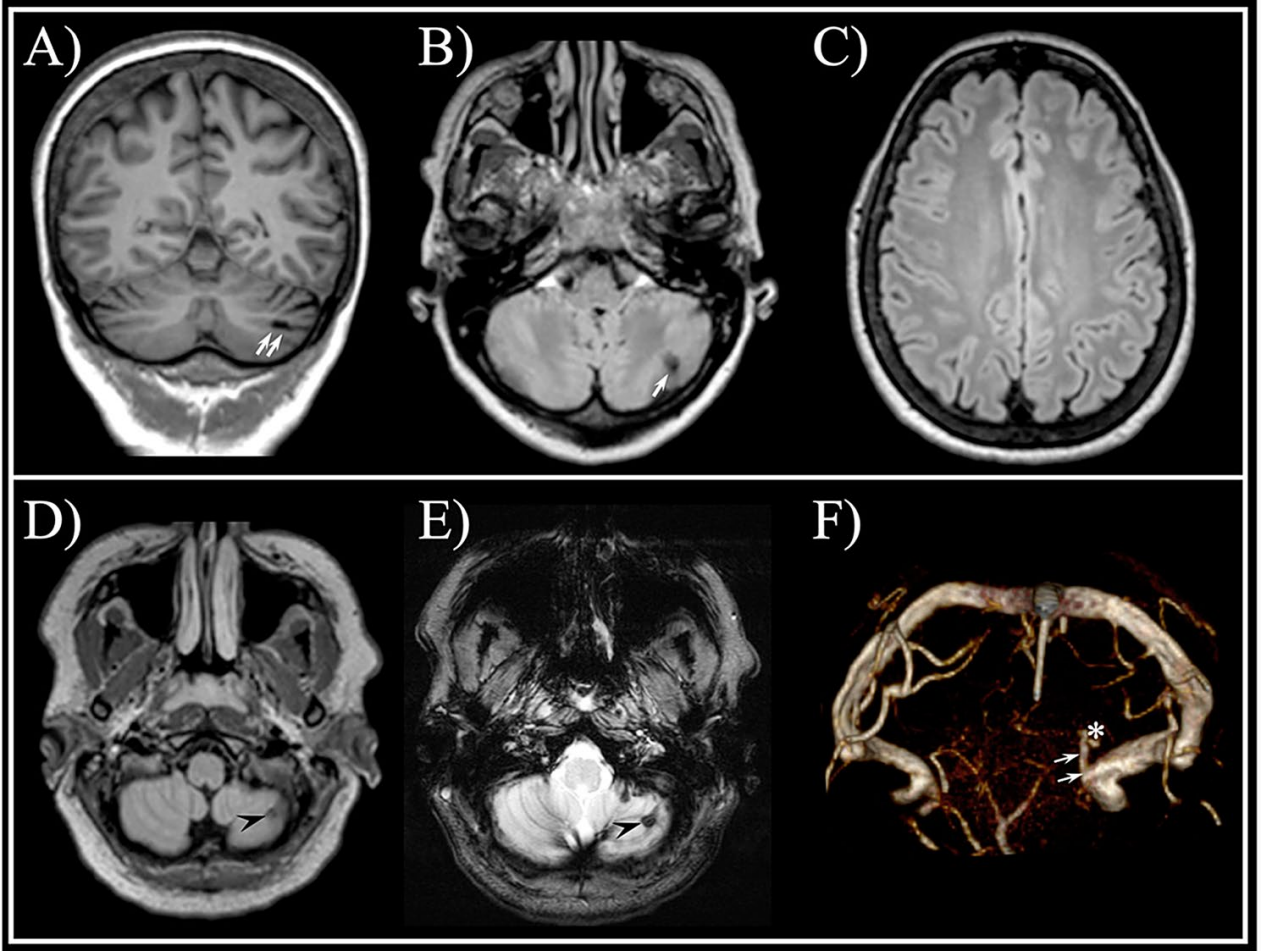
Brain mri in hereditary spherocytosis patients. Upper row images belong to a 58-year-old female: coronal T1 (**A**) and axial fluid attenuated inversion recovery (FLAIR, **B**) images show a small left cortical cerebellar infarct (white arrows); no other vascular brain lesion was detected (supratentorial flair axial image above the ventricles, **C**). Note. especially on A) that the cranial vault bone is slightly thickened due to chronic anemia. Lower row images belong to a 37-year-old female: axial flair (**D**) and T2 gradient-echo (T2*; **E**) images show a small cortical cerebellar lesion (black arrowhead) that appears markedly hypointense on T2* due to hemosiderin staining consistent with cavernous angioma; 3D-venography (**F**) shows the small venous collector originating from the lesion (*)

A 58-year-old female HS patient having mild hypercholesterolemia as sole vascular risk factor, showed a small asymptomatic left cortico-subcortical cerebellar infarct (Fig. 1. A-C), while no control had gray matter ischemic lesions (*p* = 1, Fisher exact test). Another 37-year-old female HS patient disclosed a small asymptomatic left cortico-subcortical cerebellar cavernous venous malformation (Fig. 1. D-E).

Chronic white matter lesions were found both in spherocytosis patients and controls; none presented acute ischemic lesions (i.e. hyperintense in DWI with low ADC values). Number and prevalence rate (Table 2) did not differ between patients and controls (*p* = 0.86 Wilcoxon Mann Whitney test; *p* = 1 Chi-Square test, respectively). Lesion size did not differ between patients and controls (Table 2).

**Table 2:** White matter lesion (WML) features in patients and controls

WML	Patients (%)	Controls (%)
**Prevalence Rate**	34/69 (49.3%)	28/56 (50%)
**Subjects Mean-age**	45.0±17.6	39.7±11.1
**Number**		
median	0	0.5
range	0–160	0–47
IQR	0–4	0–5.25
**Size (cm)**		
< 0.5	452/488 (92.6)	239/255 (93.7)
0.5–1.5	34/488 (7.0)	16/255 (6.3)
> 1.5	2/488 (0.4)	0
**Fazekas Score**		
0	36/69 (52.2)	28/56 (50)
1	32/69 (46.4)	28/56 (50)
2	1/69 (1.4)	0

By means of the Fazekas score, all 28 controls with white matter lesions were scored 1, while, among patients, 1/34 was scored 0, 32/34 were scored 1 and 1/34 was scored 2, showing no difference in terms of severity. Subjects with white matter lesions were significantly older than those without (45.0±17.6 vs 28.3 ± 9.8 yrs and 39.7 ± 11.1 vs 27.7 ± 6.7 yrs; *p* < 0.0001 in both patient and control groups, respectively), while age did not differ significantly between patients and controls with white matter changes (45.0±17.6 vs 39.7 ± 11.1; *p* = 0.17 t-test). Similarly, sex did not correlate with the prevalence rate of white matter changes (patients: 22/38 females vs 12/31 males; *p* = 0.17; controls: 17/36 females vs 11/20 males; *p* = 0.58). Prevalence of splenectomy did not differ between patients without or with white matter changes (25/35 vs 26/34; *p* = 0.63, Chi-Square test).

Tables 3 and 4 summarize demographic and clinical data of all Literature moya-moya and stroke patients associated with HS.

**Table 3:** Hereditary spherocytosis patients with a diagnosis of moyamoya disease: literature review

Authors	Publication year	Country	Age	Sex	Age at HS diagnosis	SPT	Age at SPT	Stroke	Transfusions	Aspirin
Holz A et al.	1998	USA	6 yrs	M	nr	nr	nr	YES	nr	nr
Tokunaga Y et al.	2001	Asia	3 yrs	M	0	YES	3 yrs	YES	YES	YES
Karvandian K et al.*	2011	Iran	10 yrs	F	1 yr	YES	nr	YES	YES	YES
Vo Van P et al.	2011	North Africa	5 yrs	F	nr	YES	5 yrs	YES	YES	YES
Gait-Carr E et al.	2017	UK	6 yrs	M	1 mo	NO		YES	NO	YES
Yadegari S et al. [6]*	2017	Iran	10 yrs	F	8 mo	YES	10 yrs	YES	YES	YES
Umesh SU et al. [5]	2018	India	9 yrs	F	5 yrs	nr	nr	YES	YES	YES
Sharma A et al. [16]	2024	India	23 yrs	M	23 yrs	NO	.-	YES	NO	NO
Sun Y et al. [17]	2024	China	17 yrs	F	17 yrs	YES	nr	YES	nr	YES

Legend: HS = Hereditary Spherocytosis; M = male; F = female; yr(s) = year(s); mo(s) = month(s); SPT = splenectomy; nr = not reported; * = may be the same case

**Table 4:** Hereditary spherocytosis patients with central nervous system infarction beyond proven moyamoya: a literature review

Authors	Year	Country	Notes	Age	Sex	Age at HS diagnosis	SPT	Age at SPT	Risk factors	Transfusion
Bruguier A et al.	1983	France	R-MCA-TS	**4 yrs**	F	0 yrs	NO	-	NO	NO
van Hilten et al. (van [15])	1989	Belgium	R-MCA-TS	66 yrs	M	nr	nr	nr	Cardiopathy	
			L-ACA-TS	65 yrs	M	nr	nr	nr	Smoking	
Schilling RF et al. [18]	1997	USA	nr	75 yrs	M	nr	YES	39 yrs	nr	nr
			nr	67 yrs	M	nr	YES	28 yrs	nr	nr
			nr	66 yrs	M	nr	YES	58 yrs	nr	nr
			nr	**50 yrs**	M	nr	YES	12 yrs	nr	nr
			nr	70 yrs	M	nr	YES	37 yrs	nr	nr
			nr	79 yrs	M	nr	YES	55 yrs	nr	nr
			nr	77 yrs	M	nr	YES	49 yrs	nr	nr
			nr	83 yrs	F	nr	YES	66 yrs	nr	nr
			nr	59 yrs	F	nr	YES	38 yrs	nr	nr
			nr	69 yrs	F	nr	YES	58 yrs	nr	nr
			nr	67 yrs	F	nr	YES	59 yrs	nr	nr
Monge-Argilés JA et al. [19]	2005	Spain	L-MCA-TS	**27 yrs**	M	7 yrs	YES	17 yrs	Factor V Leiden, smoking	nr
Ramu CS et al. [20]	2008	India	BA-TS	**38 yrs**	F	33 yrs	NO		NO	nr
Sfaihi L et al. [21]	2010	Tunisie	L-MCA-WS	**2 yrs**	F	nr	NO	-	NO	YES
Ortiz GA and Jiang H [22]	2010	USA	Abstract	58 yrs	M	nr	YES	57 yrs	after SPT: thrombocytosis, hyperlipidemia	nr
Waheed W et al. [23]	2016	USA	Spinal cord infarction	**43 yrs**	F	nr	YES	nr	diabetes	YES
Khoo TS et al. [24]	2019	Malaysia	Abstract, L-MCA-TS	**51 yrs**	M	nr	NO	-	NO	nr
Azam A et al.	2022	USA	Poster L-MCA-TS, R-hemisphere	77 yrs	F	19 yrs	YES	nr	hypertension, smoking, atrial fibrillation	nr
Ghani M et al. [25]	2023	USA	Abstract	**19 yrs**	F	nr	YES	nr	nr	nr

Legend: HS = Hereditary Spherocytosis; M = male; F = female; yr(s) = year(s); mo(s) = month(s); SPT = splenectomy; nr = not reported. R = right; L = left; MCA = middle cerebral artery; ACA = anterior cerebral artery; BA = basilar artery; TS = territorial stroke; WS = watershed stroke. Ages in bold highlight young stroke HS patients

## Discussion

The present article reports on cerebrovascular findings in a large cohort of patients with HS encompassing retrospective clinical data regarding cerebrovascular events in the young in the whole sample and prospective case-control 3T-MR data regarding intracranial parenchymal and vascular changes in a robust subset of the sample. Despite all the innuendos derived from the pre-existing Literature and the presence of potentially predisposing prothrombotic factors, such as splenectomy and/or hemolytic anemia, our findings did not reveal significant evidence of increased cerebrovascular vulnerability in HS patients. The following sections will discuss in detail the different aspects of this multifaceted study.

### Intracranial artery involvement (moyamoya disease, stenoses and aneurysms)

Hereditary spherocytosis belongs to the group of genetically determined hemolytic anemias together with sickle cell disease, alpha and beta-thalassemia, etc. Among them, the most studied is the sickle cell disease that presents a strikingly increased rate of intracranial artery stenosis. The latter results, in the most severe cases, in overt moyamoya phenomenon due to the compensatory recruitment and hypertrophy of small arterioles and the consequent characteristic “puff of smoke” at angiography. The strong relationship between sickle cell disease and moyamoya has spread the fishy opinion that also other forms of genetically determined anemia, including HS, are a possible predisposing condition for moyamoya [26].

A few case reports of moyamoya disease in children with HS (Table 3) seem to support a possible link between these conditions. Due to the high risk of cerebrovascular accidents in moyamoya, this relationship would imply an early strict monitoring of intracranial vessel status in HS, at least with non-invasive techniques such as transcranial doppler or non-contrast enhanced MR-angiography. However, moyamoya disease can be observed also in the general population and its fortuitous coexistence with HS, considering the above mentioned premises, might have acted as a driver to publish the cases. So far no study addressed this issue in a cohort of HS patients.

In our study, none of the 69 patients who had MR-angiography showed the moyamoya phenomenon or even intracranial artery stenosis. Even though the sample size might appear inadequate for excluding the relationship between HS and moyamoya, two aspects seem to clash with this association. First, we found neither intracranial artery stenosis consistent with early stages of moyamoya. Second, as moyamoya changes are irreversible and usually occur early in patients’ life (most reported cases were children ≤10 years), it appears unlikely that long persisting pathogenetic factors were not able to result in any artery change in our adult study population.

To rule out that severe cerebrovascular accidents had already wiped out moyamoya patients from our cohort, direct or phone interviews searched for stroke or sudden death at a young age ( < 55 years) in the whole sample and their families. Only a 43-year-old first-degree relative affected by HS had died for unknown cause, making the risk of underscoring the rate of moyamoya (and intracranial aneurysms, see below) rather low.

Regarding intracranial aneurysms, there is no literature data on HS. Previous studies on hemolytic anemias showed conflicting results leaving the feeling of an increased risk among some specific subgroups of patients. For example, in sickle cell disease, a high rate of intracranial aneurysms has been reported in adulthood (8%), although no control group was included [27]. A high rate (17%) was also detected in an uncontrolled study on splenectomized non-transfusion dependent beta-thalassemia patients [28] though the finding was not confirmed in a subsequent large controlled study on beta thalassemia patients [29] (9.3% with no rate differences compared to healthy controls, 8.9%). The present controlled study showed a similar rate of intracranial aneurysms in HS, showing no significant difference compared to the control group both in terms of risk of rupture (all PHASES scores ≤5) and rate (11% vs 8.9%), the latter in line with autoptic data on the general population (9.4%) [30]. Furthermore, in our whole cohort of patients (and their first-degree relatives affected by HS), no cases of subarachnoid hemorrhage have been reported. Even though we had sudden deaths of unknown cause among patients’ relatives, 2/3 were not HS patients.

Overall, literature analysis and our findings highlight how in studies on hemolytic anemias a control group is crucial to define the true pathogenic role of the disease on intracranial aneurysm formation, as aneurysms are detected also in the general population. As the present study did not reveal either a significantly increased rate of aneurysms (and intracranial artery stenosis, see above) or an increased risk of aneurysm rupture, intracranial artery examinations do not seem to be required in neurological asymptomatic HS.

### Intracranial sinus venous thrombosis

Splenectomy and hemolytic anemia are known risk factors for increased intravascular coagulation and thrombosis as they both predispose to a hypercoagulable state [31]. This should imply an increased rate of sinus venous thrombosis in HS, where splenectomy is the most common approach to contrast intravascular hemolysis. So far, only an anecdotal case of intracranial venous thrombosis has been reported [32]. Our study detected a sole case of venous sinus lumen irregularities consistent with previous thrombosis by MRI/MR-venography, and no history of previous symptomatic intracranial venous thrombosis in any of 190 HS patients. In addition, no sign of previous intracranial hemorrhagic events was found in our cohort. Our findings and the literature suggest that venous thrombotic events are rarely part of the clinical history of HS.

### Stroke, silent infarcts and white matter vascular changes

Cerebrovascular involvement in hemolytic anemias has been frequently reported both in terms of overt strokes and silent infarcts. Cases of strokes have been reported in HS as well, also independently from moyamoya vascular changes [15, 20, 23, 24, 33](Table 4). HS is supposed to cause distal small vessel occlusion (sludging syndrome) due to increased red cell aggregability, reduced red cell deformity and increased viscosity [15]. However, large vessel disease is more difficult to explain [18]. In addition, according to some authors, anemia may even represent a protective condition, at least among HS patients with no splenectomy, due to lower cholesterol blood levels and whole blood viscosity.

Analyzing HS literature, strokes in the young are strikingly frequent (16/30 considering also the HS patients with moyamoya-related strokes); however, most of them were case reports, and the association of stroke at a young age and HS might have been a prompt for publication. The sole article on an adult HS population [34] had a much lower rate of young strokes (one 50-year-old subject among 11 stroke patients in a group of 232 patients aged more than 40 years) consistent with our findings that included also pediatric patients. Indeed, in our sample, no young strokes or clinical cerebrovascular events occurred among patients or their first-degree relatives contrasting the hypothesis of a strikingly increased brain vascular vulnerability. Among those undergoing MRI, a young patient revealed a small asymptomatic cerebellar infarction. Even though the finding did not reach significance, the issue remains an open question. Once again, the presence of some cases of sudden death of unknown causes among patients’ relatives might raise the suspicion of undiagnosed severe stroke. However, two out of the three cases had no HS. Future large studies will help define if the risk of brain infarction, at least asymptomatic, should prompt for prevention or monitoring.

Regarding white matter changes, our study seems to highlight two important aspects. The first aspect concerns the impact of splenectomy on white matter involvement. While an increased risk of stroke after splenectomy has been shown [35, 36], so far, only two uncontrolled studies on thalassemia intermedia have found a high rate of white matter changes in splenectomized patients. This finding was not confirmed in other studies on beta-thalassemia patients and, most importantly, no literature data are available on HS. In our study, splenectomy did not result as a risk factor of increased white matter involvement in HS, challenging its role in small vessel disease brain involvement.

Second, HS does not seem to carry an increased microvascular vulnerability compared to healthy subjects, as the two groups did not differ in terms of prevalence rate, number and size of white matter lesions.

This finding further strengthens the near absence of early clinical and imaging cerebrovascular events in our study as white matter lesions and brain infarcts share most of the pathogenic mechanisms. Actually, in HS reduced red cell deformability and increased blood viscosity should primarily result in distal small vessel occlusion (sludging syndrome) with chronic parenchymal ischemia and diffuse white matter changes rather than with large overt territorial infarctions. On the other side, white matter hyperintensities are a known independent risk factor for cerebrovascular events [37], as silent cerebral ischemic lesions cause a threefold increase of future stroke [38]. According to our MRI findings, this risk factor appears to be equally present among HS patients and healthy controls (49.3% vs 50%).

## Limits

This study has some limits to be taken into account. First, being a rare disease the sample is relatively small, especially considering those who had undergone MRI and MR-angiography investigations. The sample size implies that some conclusions, especially those highlighting the lack of a significant cerebrovascular vulnerability in HS, might benefit from validatory studies. Second, data on the cerebrovascular history of the whole sample of patients was based on interviews and might be less precise than regular monitoring. A long-term disease-specific registry could ameliorate data collection on cerebrovascular events thus strengthening our preliminary observations. Third, this is a single center-based study and is therefore representative of this region. A multicenter prospective study will likely reveal if our results can be generalized.

## Conclusion

Cerebrovascular involvement in young hereditary spherocytosis subjects does not seem to differ from the general population. In fact, the present clinical and MRI study did not reveal 1) overt moyamoya disease or intracranial artery stenosis suspicious for initial moyamoya disease; 2) significantly increased prevalence rate of intracranial aneurysms; 3) increased prevalence or severity of vascular-like white matter lesions; 4) convincing elements of early cerebrovascular involvement. Even though validatory studies are required, according to our study, a strict neurological and MR monitoring (either with conventional MRI or MR angiography sequences) in neurologically asymptomatic HS patients does not seem nowadays to be recommended.

## Data Availability

Data is available at request to the corresponding author.

## Abbreviations

HS: Hereditary spherocytosis
MRI: Magnetic resonance imaging
WML: White matter lesions
FLAIR: Fluid attenuated inversion recovery
DWI: Diffusion weighted imaging
ADC: Apparent diffusion coefficient
